# Unplanned Surgery After Transcatheter Closure of Ventricular Septal Defect in Children: Causes and Risk Factors

**DOI:** 10.3389/fped.2021.772138

**Published:** 2021-11-30

**Authors:** Penghui Yang, Zhijun Wu, Zhiyuan Liu, Jing Zhang, Hao Zhou, Xiaojuan Ji, Qijian Yi, Mi Li

**Affiliations:** ^1^Department of Cardiovascular Medicine, Children's Hospital of Chongqing Medical University, Chongqing, China; ^2^National Clinical Research Center for Child Health and Disorders, Chongqing, China; ^3^Ministry of Education Key Laboratory of Child Development and Disorders, Chongqing, China; ^4^Chongqing Key Laboratory of Pediatrics, Chongqing, China; ^5^Department of Pediatrics, Chongqing Iron and Steel General Hospital, Chongqing, China; ^6^Department of Cardiology, The Second Affiliated Hospital of Chongqing Medical University, Chongqing, China; ^7^Department of Ultrasound Investigations, Children's Hospital of Chongqing Medical University, Chongqing, China

**Keywords:** ventricular septal defect, transcatheter closure, unplanned surgery, causes and risk factors, children

## Abstract

**Objectives:** To evaluate the causes and risk factors of unplanned surgery after transcatheter closure of ventricular septal defect (VSD) in children.

**Methods:** A total of 773 patients with VSD who had the devices transcatheter released between January 2013 and December 2018 in our institution were retrospectively reviewed. Univariate and multivariate analyses were used to identify the risk factors for unplanned surgery.

**Results:** Twenty four patients (3.1%) underwent unplanned surgery after transcatheter closure of VSD. The most common cause for unplanned surgery was new-onset or worsening aortic regurgitation (14/24; 58.3%), followed by occluder migration (4/24; 16.7%), complete atrioventricular block (2/24; 8.3%), severe hemolysis (2/24; 8.3%), residual shunt (1/24; 4.2%), and occluder edge near the tricuspid valve chordae (1/24; 4.2%). Logistic regression analysis revealed that primary aortic valve prolapse (OR: 5.507, 95%CI: 1.673–18.123, *P* = 0.005); intracristal VSD (OR: 8.731, 95%CI: 2.274–33.527, *P* = 0.002); eccentric occluder (OR: 4.191, 95%CI: 1.233–14.246, *P* = 0.022); larger occluder size (OR: 1.645, 95%CI: 1.331–2.033, *P* < 0.001); and pulmonary artery systolic pressure ≥45 mmHg (OR: 4.003, 95%CI: 1.073–14.941, *P* = 0.039) were risk factors for unplanned surgery.

**Conclusions:** New-onset or worsening aortic regurgitation was the primary cause for unplanned surgery after transcatheter closure of VSD in children. Primary aortic valve prolapse, intracristal VSD, eccentric occluder, larger occluder size, pulmonary artery systolic pressure ≥45 mmHg could increase the risk of unplanned surgery.

## Introduction

Ventricular septal defect (VSD) is the most common form of congenital heart diseases in children, occurring at a rate of ~3 per 1,000 live births (1). Surgical closure has been regarded as the standard treatment strategy. However, since Lock et al. (2) described transcatheter closure of VSD with the Rashkind double umbrella device in 1988, techniques and devices for transcatheter treatment have been continuously evolved and refined in the past decades (3, 4). Currently, transcatheter closure of VSD has been accepted as a valuable alternative to surgical treatment for certain types of VSD, with the benefit of shorter hospitalization time and absence of sternotomy and extracorporeal circulatory support (5, 6). Increasing attention has been paid to the adverse events associated with the transcatheter approach, and a series of studies have reported that its short—and long-term complications are acceptable (7–9). However, unplanned surgery after transcatheter closure has not been well-described. The purpose of this study was to evaluate the causes and corresponding risk factors of unplanned surgery after transcatheter closure of VSD in children in a single-center cohort.

## Methods

### Patients

Clinical data of patients with VSD who underwent transcatheter device closure in our institution were retrospectively reviewed from January 2013 to December 2018. All patients fulfilled the following criteria: (1) age <18 years old, (2) VSD diagnosed by standard transthoracic echocardiography (TTE) pre-procedurally, (3) the severity of aortic regurgitation and aortic valve prolapse before the procedure not greater than mild, and (4) intra-procedural implantation of the occluder. Exclusion criteria were: (1) transcatheter closure for residual shunt after VSD surgical repair, and (2) presence of uncontrolled infection. According to whether unplanned surgery was performed, patients were assigned into the unplanned surgery group and the control group. Unplanned surgery refers to surgery performed after the release of the occluder due to new or worsening problems. The study was approved by the Ethics Committee of the Children's Hospital of Chongqing Medical University and complied with the Declaration of Helsinki.

### Transcatheter Procedure and Follow-Up

The transcatheter procedure was performed following standard domestic guidelines (10). Briefly, heparin was given 100 U/kg during the procedure. Standard left and right cardiac catheterization, left ventriculography, and ascending aorta angiography were performed. An arteriovenous circuit was established through a femoral vein approach. A long sheath was advanced to the position above the aortic valve through the arteriovenous circuit. The delivery sheath was then retrieved into the left ventricle and directed to the apex. The selected device was deployed through the long sheath under fluoroscopic control. The device was completely released when the TTE result was satisfactory. Finally, repeated TTE, left ventriculography, and ascending aortic angiography were performed to assess appropriate device position, residual shunt, and valvular regurgitation. If problems were discovered during the repeated examinations, such as significant aortic regurgitation, occluder migration, complete atrioventricular block (cAVB), and significant residual shunt even using the large-sized occluder, we would attempt to retrieve the occluder percutaneously. When significant aortic regurgitation occurred, we would remove the occluder even if there was no clear evidence of an association with valve impingement because of the potential risk of the valve interference, especially when the edge of the occluder was close to the aortic valve.

**Figure 1:**
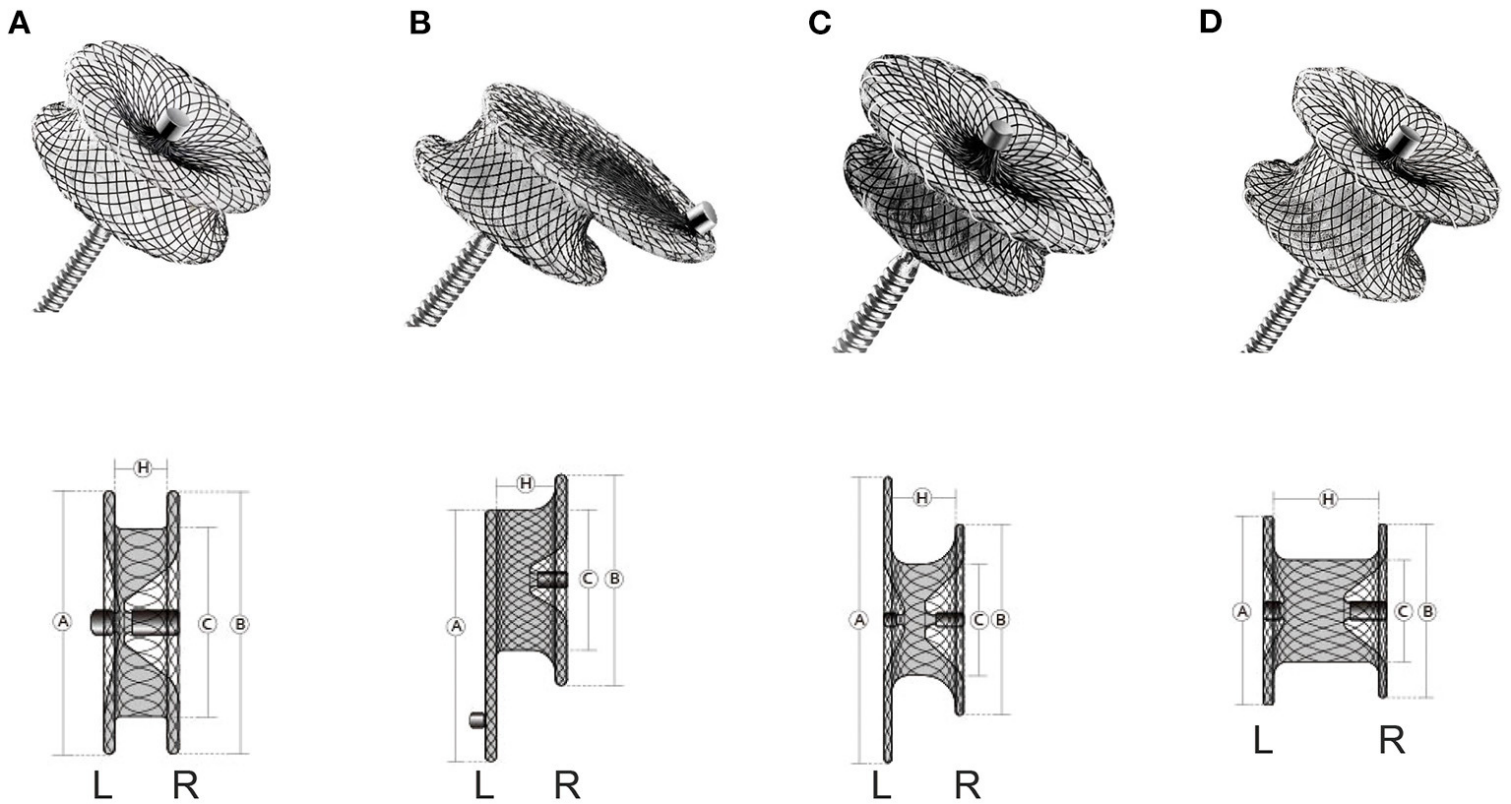
The various occluders and corresponding schematic diagrams. **(A)**, symmetric occluder. **(B)**, eccentric occluder. **(C)**, small-waist occluder. **(D)**, muscular occluder. L, left; R, right.

The devices used in our study were the modified double-disk VSD occluders (Lifetech Scientific, Shenzhen, China; Shanghai Shape Memory Alloy, Shanghai, China; Starway Medical, Beijing, China). Four subtypes of modified double-disk occluders were used in this study: symmetrical occluder, eccentric occluder, small-waist occluder, and muscular occluder (any of the three companies could manufacture the four subtypes of modified double-disk occluders). All four subtypes of occluders have the same right disk, whose diameter is 4 mm larger than the waist. In the symmetric and muscular occluder, the left disk is symmetric to the right disk, and the diameter of the left disk is 4 mm larger than the waist. In the eccentric occluder, the left disk exceeds the waist by 0 mm on its aortic side and by 6 mm on the opposite side. In the small-waist occluder, the diameter of the left disk is 8 mm larger than the waist. The waist length of the symmetrical, eccentric, and small-waist occluder is 3–4 mm. However, the waist length of the muscular occluder is 5 or 7 mm (Figure 1).

Patients were discharged from the hospital 5–6 days after the procedure if there were no adverse events. All patients received aspirin (3–5 mg/kg orally daily) for 6 months. Follow-up was performed at 1, 3, 6, and 12 months after the procedure, and yearly thereafter. The follow-up contents included clinical examination, electrocardiogram, and TTE.

### Data Collection

We collected the following information from the hospital's electronic medical record system: (1) demographic information: age, sex, weight (2) preoperative data: VSD size, VSD type, whether combined with arrhythmia, whether combined with aortic regurgitation, whether combined with primary aortic valve prolapse (3) intra- and post-operative data: VSD size, pulmonary artery systolic pressure (PASP), pulmonary artery mean pressure (PAMP), occluder size, occluder type, sheath in-out time, residual shunt, arrhythmia, aortic regurgitation, tricuspid regurgitation, hemolysis, occluder migration, and whether unplanned surgery was performed.

### Statistical Analysis

Continuous variables were expressed as mean ± SD or median and interquartile range and categorical variables were expressed as number and percentage. The Shapiro-Wilk test was used to verify whether the variables were normally distributed. To compare the differences between the two groups, the independent *t*-tests were used for normally distributed continuous variables, Mann–Whitney *U*-tests for non-normally distributed continuous variables, and chi-square tests or Fisher exact tests for categorical variables. Univariate and multivariate logistic regression analyses were performed to investigate the risk factors associated with unplanned surgery after transcatheter closure of VSD. Factors with statistical significance by univariate regression analysis were further analyzed by multivariate regression, and the forward LR method was used. Finally, the statistically significant factors were considered as independent risk factors. PAMP and PASP were converted to categorical variables according to clinically meaningful cut-off values before logistic regression analysis. All statistical tests were two-sided, and *P* < 0.05 was considered significant. All statistical analyses were performed with SPSS version 22.0 (SPSS, Chicago, IL).

## Results

Based on the inclusion and exclusion criteria, a total of 773 patients were included in the study. During a median follow-up of 5 years, 24 patients (3.1%) underwent unplanned surgery, all of which were performed at a median of 4 days (IQR 3–5 days) after the transcatheter procedure. The characteristics of the entire cohort are shown in Table 1.

**Table 1:** General characteristics.

**Variable**	**Total (*n* = 773)**	**Control (*n* = 749)**	**Unplanned surgery (*n* = 24)**	***P*** **-value**
Age (month)	45.1 ± 26.3	45.1 ± 26.5	45.1 ± 20.9	0.999
Male, *n* (%)	397 (51.4)	383 (51.1)	14 (58.3)	0.487
Weight (kg)	15.5 ± 5.8	15.5 ± 5.8	15.3 ± 4.6	0.863
Preoperative arrhythmias, *n* (%)	540 (69.9)	527 (70.4)	13 (54.2)	0.095
Preoperative aortic regurgitation, *n* (%)	80 (10.3)	70 (9.3)	10 (41.7)	<0.001
Primary aortic valve prolapse, *n* (%)	147 (19.0)	132 (17.6)	15 (62.5)	<0.001
VSD type, *n* (%)				<0.001
Perimembranous VSD	747 (96.6)	731 (97.6)	16 (66.6)	
Intracristal VSD	22 (2.8)	15 (2.0)	7 (29.2)	
Muscular VSD	4 (0.5)	3 (0.4)	1 (4.2)	
VSD size (mm) (angiography)	4.7 ± 2.9	4.6 ± 2.9	6.2 ± 2.3	0.030
PASP (mmHg)	29 (24, 34)	28 (24, 34)	34 (27, 44)	<0.001
PAMP (mmHg)	17 (14, 21)	17 (14, 21)	21 (17, 26)	0.023
Occluder type, *n* (%)				<0.001
Symmetric occluder	707 (91.5)	697 (93.1)	10 (41.7)	
Eccentric occluder	54 (7.0)	42 (5.6)	12 (50.0)	
Small-waist occluder	8 (1.0)	7 (0.9)	1 (4.2)	
Muscular occluder	4 (0.5)	3 (0.4)	1 (4.2)	
Occluder size (mm)	6.6 ± 1.6	6.5 ± 1.5	9.2 ± 2.4	<0.001
Sheath in-out time (min)	40 (30, 55)	40 (30, 50)	60 (50, 73)	<0.001

*Data are mean ± SD, median (interquartile range), or n (%). P-values comparing Control and Unplanned surgery were from the 2-sample t-test, χ^2^ test, Fisher exact test, or Mann-Whitney U-test. P < 0.05 was considered statistically significant. VSD, ventricular septal defect; PASP, pulmonary artery systolic pressure; PAMP, pulmonary artery mean pressure*.

**Figure 2:**
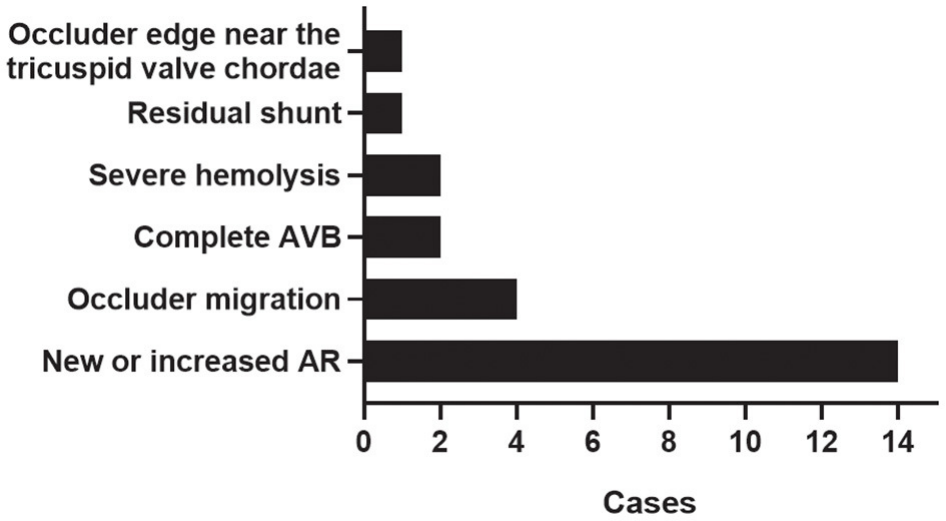
Causes for unplanned surgery. AVB, complete atrioventricular block; AR, aortic regurgitation.

Among the causes of unplanned surgery, new or increased aortic regurgitation ranked first, accounting for 58.3% (14/24), followed by occluder migration at 16.7% (4/24), cAVB at 8.3% (2/24), severe hemolysis at 8.3% (2/24), residual shunt at 4.2% (1/24), and occluder edge near the tricuspid valve chordae at 4.2% (1/24) (Figure 2). Five unplanned surgical procedures occurred after early successful transcatheter closure procedures, all of which occurred in patients with perimembranous VSD. A 3-year-old girl (VSD size: 9 mm; device used: 10 mm symmetric occluder) developed cAVB 24 h after transcatheter closure and did not get improved after intravenous methylprednisolone. She underwent open heart surgery and the heart returned to sinus rhythm after removing the device. Two 2-year-old girls (VSD size: 10 and 8.7 mm, respectively; devices used: 12 mm symmetric occluder and 11 mm eccentric occluder, respectively) experienced severe hemolysis after transcatheter closure. Both of them received sodium bicarbonate, methylprednisolone, blood transfusion, fluid rehydration therapy, and removal of the device as well as the reparation of VSD under cardiopulmonary bypass 48 h after the transcatheter procedure. A 3-year-old girl (VSD size: 9.1 mm; device used: 11 mm symmetric occluder) and a 4-year-old boy (VSD size 6.9 mm; device used: 10 mm symmetric occluder) underwent device removal and VSD repair under cardiopulmonary bypass on day 3 after transcatheter closure because of new onset and worsening aortic regurgitation, respectively. The remaining unplanned surgical procedures occurred in the following situations: problems were discovered during the repeated examinations performed immediately after the release of the device, the device was subsequently retrieved percutaneously, and finally, the patient was sent for surgery (Table 2).

**Table 2:** Demographics and clinical data of VSD patients who underwent unplanned surgery.

**Case**	**Age (month)**	**Weight (kg)**	**Pre-operative arrhythmias**	**Pre-operative AR**	**Primary AVP**	**VSD type**	**VSD size (TTE/angio) (mm)**	**Occluder type/size (mm)**	**PASP (mmHg)**	**PAMP (mmHg)**	**Event**
1	87	18.5	Sinus arrhythmia	None	None	Intracristal	7.0/5.8	Eccentric/11	40	21	A
2	24	12.0	None	Yes	Yes	Intracristal	5.9/3.5	Eccentric/6	24	17	A
3	94	27.0	IRBBB	Yes	Yes	Intracristal	5.6/2.5	Eccentric/5	28	16	A
4	27	12.0	None	None	None	Perimembranous	7.7/5.6	Symmetric/7	24	17	A
5	69	21.0	Sinus arrhythmia	None	Yes	Intracristal	8.0/5.0	Symmetric/6	30	21	A
6	27	13.5	None	Yes	Yes	Perimembranous	5.7/5.0	Eccentric/8	33	18	A
7	26	10.0	None	None	Yes	Perimembranous	3.7/3.3	Eccentric/6	32	17	A
8	37	14.0	IRBBB	None	None	Perimembranous	10.0/9.1	Symmetric/11	38	25	A
9	36	11.5	None	Yes	Yes	Perimembranous	7.4/5.6	Eccentric/10	54	35	A
10	60	21.5	Sinus arrhythmia	Yes	Yes	Intracristal	4.8/5.0	Eccentric/10	30	23	A
11	36	15.5	Sinus arrhythmia	Yes	Yes	Perimembranous	2.7/2.0	Eccentric/5	23	11	A
12	34	11.5	Sinus arrhythmia	Yes	Yes	Perimembranous	9.6/6.4	Eccentric/8	23	12	A
13	52	15.0	None	None	Yes	Perimembranous	8.3/6.9	Symmetric/10	40	23	A
14	39	14.0	None	Yes	Yes	Perimembranous	5.6/4.5	Symmetric/9	26	16	A
15	37	16.0	Sinus arrhythmia	None	None	Perimembranous	11.3/8.2	Symmetric/12	60	44	B
16	63	19.0	None	None	None	Perimembranous	8.0/8.7	Small-waist/9	35	20	B
17	84	23.0	None	None	Yes	Intracristal	7.0/5.0	Eccentric/10	25	14	B
18	34	12.0	IRBBB	None	Yes	Perimembranous	7.9/6.9	Eccentric/12	61	45	B
19	38	15.0	None	None	None	Perimembranous	9.6/9.0	Symmetric/10	39	25	C
20	60	20.0	Sinus arrhythmia	Yes	Yes	Perimembranous	6.4/6.0	Symmetric/8	33	20	C
21	29	10.0	Sinus arrhythmia	None	None	Perimembranous	12.0/10.0	Symmetric/12	67	48	D
22	28	11.0	Sinus arrhythmia	Yes	Yes	Perimembranous	10.7/8.7	Eccentric/11	43	23	D
23	30	12.0	Sinus tachycardia	None	None	Intracristal	8.0/5.8	Symmetric/10	45	27	E
24	32	11.0	None	None	None	Muscular	8.8/9.7	Muscular/14	48	30	F

*VSD, ventricular septal defect; AR, aortic regurgitation; AVP, aortic valve prolapse; TTE, transthoracic echocardiography; PASP, pulmonary artery systolic pressure; PAMP, pulmonary artery mean pressure; IRBBB, incomplete right bundle-branch block; Event A, new or increased aortic regurgitation; Event B, occluder migration; Event C, complete atrioventricular block; Event D, severe hemolysis; Event E, residual shunt; Event F, occluder edge near the tricuspid valve chordae*.

In our patients who underwent unplanned surgery due to aortic regurgitation, primary aortic valve prolapse and intracristal VSD were present in 12 cases and absent in only two cases. During the transcatheter procedure performed on these 12 patients, six patients experienced repeated press of the delivery sheath, three patients had right coronary valve interference by the left disk of the occluder, and three patients had repeated adjustment of the eccentric occluder. The remaining two cases of aortic regurgitation occurred in a 2-year-old boy after placement of a 7 mm symmetric occluder to occlude a 5.6 mm perimembranous defect and in a 3-year-old girl after placement of an 11 mm symmetric occluder to occlude a 9.1 mm perimembranous defect. The mechanism of aortic regurgitation in these two patients may be associated with the impingement of the occluder on the valve leaflets.

Compared with the control group, more patients who underwent unplanned surgery had a higher proportion of preoperative aortic regurgitation (41.7 vs. 9.3%), a higher proportion of primary aortic valve prolapse (62.5 vs. 17.6%), a higher proportion of intracristal VSD (29.2 vs. 2.0%), a higher proportion of eccentric occluder (50.0 vs. 5.6%), larger VSD size, higher PASP, larger occluder size, and longer sheath in-out time (all *P* < 0.001). Patients who did not undergo unplanned surgery had a higher proportion of perimembranous VSD (97.6 vs. 66.6%) and a higher proportion of symmetric occluder (93.1 vs. 41.7%) than patients who underwent unplanned surgery (all *P* < 0.001).

The results of univariate and multivariate logistic regression analyses are shown in Table 3. In univariate analysis, preoperative aortic regurgitation; primary aortic valve prolapse; intracristal VSD; VSD size; PASP ≥45 mmHg; PAMP ≥25 mmHg; eccentric occluder; occluder size; and sheath in-out time were associated with unplanned surgery. In multivariate analysis, we found that primary aortic valve prolapse (OR: 5.507, 95%CI: 1.673–18.123, *P* = 0.005); intracristal VSD (OR: 8.731, 95%CI: 2.274–33.527, *P* = 0.002); eccentric occluder (OR: 4.191, 95%CI: 1.233–14.246, *P* = 0.022); larger occluder size (OR: 1.645, 95%CI: 1.331–2.033, *P* < 0.001); and PASP ≥45 mmHg (OR: 4.003, 95%CI: 1.073–14.941, *P* = 0.039) were associated with increased odds of unplanned surgery after transcatheter closure of VSD in children.

**Table 3:** Univariate and multivariate logistic analyses of risk factors for unplanned surgery after transcatheter closure of VSD.

**Factor**	**Univariate analysis**		**Multivariate analysis**	
	**OR (95%CI)**	***P*** **-value**	**OR (95%CI)**	***P*** **-value**
Age (month)	1.000 (0.985–1.016)	0.999	–	–
Male	1.338 (0.587–3.050)	0.489	–	–
Weight (kg)	0.994 (0.923–1.069)	0.863	–	–
Preoperative arrhythmias	0.498 (0.220–1.128)	0.095	–	–
Preoperative aortic regurgitation	6.929 (2.967–16.178)	<0.001	–	–
Primary aortic valve prolapse	7.790 (3.338–18.181)	<0.001	5.507 (1.673–18.123)	0.005
Intracristal VSD	20.149 (7.281–55.759)	<0.001	8.731 (2.274–33.527)	0.002
VSD size (mm) (angiography)	1.095 (1.009–1.189)	0.030	–	–
PASP ≥45 (mmHg)	7.299 (2.862–18.611)	<0.001	4.003 (1.073–14.941)	0.039
PAMP ≥25 (mmHg)	3.283 (1.369–7.872)	0.008	–	–
Eccentric occluder	16.833 (7.133–39.723)	<0.001	4.191 (1.233–14.246)	0.022
Occluder size (mm)	1.759 (1.464–2.115)	<0.001	1.645 (1.331–2.033)	<0.001
Sheath in-out time (min)	1.031 (1.015–1.046)	<0.001	–	–

*VSD, ventricular septal defect; PASP, pulmonary artery systolic pressure; PAMP, pulmonary artery mean pressure; OR, odds ratio*.

## Discussion

Transcatheter closure of specific types of VSD has been widely performed, especially in developing countries, with encouraging follow-up results (11). Nevertheless, unplanned surgery may occur, which requires sufficient attention. To our knowledge, this is the first detailed study to describe unplanned surgery after transcatheter closure of VSD in children.

The present study showed that 3.1% of patients (24/773) underwent unplanned surgery after successful attempt of transcatheter VSD closure. In our cohort, the most common cause of unplanned surgery was new-onset or worsening aortic regurgitation (14/24).

Aortic regurgitation implies aortic valve incompetence with a risk of developing left ventricular dysfunction, heart failure, and even death (12). Aortic regurgitation is one of the major considerations in the transcatheter closure of VSD (13). Surgery performed because of aortic regurgitation that occurred during or after the transcatheter procedure has been reported (13, 14). In our study, 14 patients underwent unplanned surgery due to aortic regurgitation after occluder implantation, accounting for 58.3% of the total number of unplanned surgery. Therefore, it is important to pay enough attention to aortic regurgitation.

The possible cause of aortic regurgitation is the edema or damage of the aortic valve caused by the guidewire, delivery sheath, or occluder, especially in patients with intracristal VSD or primary aortic valve prolapse (15). Aortic valve prolapse is intimately linked to the occurrence or worsening of aortic regurgitation (16). This is because the distal part of the delivery sheath is difficult to press into the left ventricle, and the process of repeatedly pressing the distal part of the delivery sheath into the left ventricle and establishing arteriovenous track would increase the risk of aortic valve injury. Furthermore, if the distal part of the delivery sheath fails to press into the left ventricle, the left disc of the occluder will be released in the ascending aorta, thereby increasing the possibility of aortic valve damage. Most importantly, significant aortic regurgitation after device implantation may be related to the failure to accurately estimate the size and location of the defect due to prolapse of the aortic valve into the defect site. The left disk of the larger sized occluder may interfere with the right coronary valve and cause aortic regurgitation. For patients with intracristal VSD, the location of the defect is high, the upper rim of the defect is adjacent to the aortic valve. Even though the eccentric occluder is preferred in these patients to avoid aortic regurgitation, aortic regurgitation still occasionally occurs. The underlying mechanism may include the following. Objectively, the deployment of the eccentric occluder is technically difficult, because it is necessary to ensure that the eccentric surface of the occluder keeps toward the apex. The repeated adjustment process undoubtedly increases the possibility of aortic valve injury. Additionally, the unextended left disk of the eccentric occluder toward the aorta forms a wider platform, and the junction of the aortic root and valve body is supported by the platform, which may affect valvular activity. Finally, intracristal VSD often coexists with aortic prolapse, which also leads to an increased chance of aortic injury.

At present, transcatheter occlusion for patients with aortic prolapse or intracristal VSD is constantly being tried (15, 17–19), while the efficacy and safety remain controversial. By reviewing patients with prolapse and intracristal VSD in our cohort, we summarized the following experiences. The delivery sheath with a curved and soft distal part can be easier pressed into the left ventricle. Secondly, left ventriculography at 60–90°left anterior oblique and 20–30° cranial projection can clearly show the relationship between the defect and the aortic valve. The left ventricular angiography performed after establishing the arteriovenous track and placing the sheath can better reflect the size of the defect because the sheath holds up the aortic valve. Of course, the measurements from various views by TTE and the diameter of the sheath can help to evaluate the size of the defect. Finally, to make the “0” edge of the occluder oriented toward the aorta, the long edge should point to the 5–7 o'clock direction when the eccentric occluder is inserted into the long sheath.

Occluder migration and significant residual shunt caused by the smaller sized occluder; cAVB and the possibility of tricuspid chordae rupture caused by the larger sized occluder; and severe hemolysis caused by the larger defect, larger sized occluder, persistent residual shunt, and higher pulmonary artery pressure also accounted for unplanned surgery in our patients. Thus, selecting the appropriate device is crucial.

Recognizing the associated risk factors will be helpful for better prevention for the occurrence of unplanned surgery. Our univariate and multivariate analyses showed the association between unplanned surgery and multiple risk factors, including primary aortic valve prolapse, intracristal VSD, eccentric occluder, occluder size, and PAS ≥ 45 mmHg.

As mentioned in the preceding text, primary aortic valve prolapse, intracristal VSD, and eccentric occluder are related to aortic regurgitation. In addition, because the intracristal VSD is usually partially covered by the right coronary valve, the true size of the defect is usually underestimated, resulting in the small occluder (the vast majority are eccentric occluder) being selected, causing residual shunt and even occluder migration (17). According to the experience of our center, for these patients, the occluder selected for closure could be 2–4 mm larger than the defect diameter measured by angiography. If the defect was ≥ 5 mm, the occluder that was 4–6 mm larger than the maximum size of the defect measured by angiography could be chosen.

Our study indicated that larger occluder size was associated with unplanned surgery. The large-sized occluders were associated with postprocedural arrhythmia, which has been reported by previous studies (20, 21). The larger the size of the occluder, the more serious compression of the ventricular septum and conduction system. In our series, the occluder size of the 2 patients who underwent unplanned surgery for cAVB was 8 and 10 mm, respectively. Further, the larger the occluder, the closer its edge is to the valve, which may result in valve regurgitation (22).

The presence of massive intracardiac left-to-right shunt causes an overflow in the pulmonary circulation. Bergersen et al. (23) found that elevated PASP was a risk factor for adverse events related to transcatheter procedures. Similarly, our study showed that PASP ≥ 45 mmHg was an independent risk factor for unplanned surgery. Therefore, intervention before PASP ≥ 45 mmHg in VSD patients may reduce the occurrence of unplanned surgery, whereas, for VSD patients with excessive PASP, initial pharmacological reduction of pulmonary artery pressure followed by transcatheter therapy could be considered.

Some limitations of the present study should be acknowledged. First, it was a single-center retrospective study. This not only lead to restrictions in the sample size, but it also limited the generalizability of the findings. Second, our analysis of risk factors for unplanned surgery was also limited because we only explored preoperative and selected intraoperative factors. As such, additional intraoperative factors and postoperative management may contribute to the prognosis of patients.

## Conclusion

In summary, we found that the rate of unplanned surgery in our center after transcatheter closure of VSD in children was 3.1%, and the main cause of unplanned surgery was new-onset or worsening aortic regurgitation. Risk factors for unplanned surgery include primary aortic valve prolapse, intracristal VSD, eccentric occluder, larger occluder size, and PASP ≥ 45 mmHg.

## Data Availability Statement

The original contributions presented in the study are included in the article/supplementary materials, further inquiries can be directed to the corresponding author/s.

## Ethics Statement

The studies involving human participants were reviewed and approved by Ethics Committee of the Children's Hospital of Chongqing Medical University. Written informed consent to participate in this study was provided by the participants' legal guardian/next of kin. Written informed consent was obtained from the individual(s), and minor(s)' legal guardian/next of kin, for the publication of any potentially identifiable images or data included in this article.

## Author Contributions

PY, ZW, ZL, and ML: study design. ZW, ZL, and JZ: data collection. PY, ZW, ZL, JZ, and HZ: analysis and interpretation of data. PY and ZW: drafting of the manuscript. XJ, QY, and ML: critical revision of the manuscript for important intellectual content. All authors contributed to the article and approved the submitted version.

## Funding

This study was supported by the Key Project of Medical Research Program of Chongqing Municipal Health Committee (2016ZDxm018).

## Conflict of Interest

The authors declare that the research was conducted in the absence of any commercial or financial relationships that could be construed as a potential conflict of interest.

## Publisher's Note

All claims expressed in this article are solely those of the authors and do not necessarily represent those of their affiliated organizations, or those of the publisher, the editors and the reviewers. Any product that may be evaluated in this article, or claim that may be made by its manufacturer, is not guaranteed or endorsed by the publisher.
